# Shotgun Metagenomic Sequencing Reveals Functional Genes and Microbiome Associated with Bovine Digital Dermatitis

**DOI:** 10.1371/journal.pone.0133674

**Published:** 2015-07-20

**Authors:** Martin Zinicola, Hazel Higgins, Svetlana Lima, Vinicius Machado, Charles Guard, Rodrigo Bicalho

**Affiliations:** Department of Population Medicine and Diagnostic Sciences, Cornell University, Ithaca, New York, United States of America; GI Lab, UNITED STATES

## Abstract

Metagenomic methods amplifying 16S ribosomal RNA genes have been used to describe the microbial diversity of healthy skin and lesion stages of bovine digital dermatitis (DD) and to detect critical pathogens involved with disease pathogenesis. In this study, we characterized the microbiome and for the first time, the composition of functional genes of healthy skin (HS), active (ADD) and inactive (IDD) lesion stages using a whole-genome shotgun approach. Metagenomic sequences were annotated using MG-RAST pipeline. Six phyla were identified as the most abundant. *Firmicutes* and *Actinobacteria* were the predominant bacterial phyla in the microbiome of HS, while *Spirochetes*, *Bacteroidetes* and *Proteobacteria* were highly abundant in ADD and IDD. *T*. *denticola-like*, *T*. *vincentii-like* and *T*. *phagedenis-like* constituted the most abundant species in ADD and IDD. Recruitment plots comparing sequences from HS, ADD and IDD samples to the genomes of specific *Treponema* spp., supported the presence of *T*. *denticola* and *T*. *vincentii* in ADD and IDD. Comparison of the functional composition of HS to ADD and IDD identified a significant difference in genes associated with motility/chemotaxis and iron acquisition/metabolism. We also provide evidence that the microbiome of ADD and IDD compared to that of HS had significantly higher abundance of genes associated with resistance to copper and zinc, which are commonly used in footbaths to prevent and control DD. In conclusion, the results from this study provide new insights into the HS, ADD and IDD microbiomes, improve our understanding of the disease pathogenesis and generate unprecedented knowledge regarding the functional genetic composition of the digital dermatitis microbiome.

## Introduction

Bovine digital dermatitis (DD) is a prevalent disease that affects cattle worldwide [1–3]. It is associated with reduced milk production, decreased reproductive performance, high treatment cost and increased risk of culling, resulting in significant economic losses [4–7]. Current disease prevention and control methods include foot bathing using copper sulfate (CuSO4), formalin or antibiotic solution [8–10]. Additionally, commercial products containing zinc sulfate (ZnSO4) and a variety of other disinfectants (e.g. glutaraldehyde and hydrogen peroxide) are available; however published clinical trials evaluating their efficacy are limited.

It has been reported that a variety of bacteria are associated with DD, including Treponema spp., Fusobacterium necrophorum, Porphyromonas spp., Bacteroides spp., Campylobacter spp., Guggenheimella spp., Borrelia spp., Dichelobacter nodosus and Candidatus Aemobophilus asiaticus [11–16]. However, multiple studies using different molecular techniques have investigated the role of Spirochetes, particularly Treponema spp., which are considered to be the most important pathogen involved with DD [15–18]. Markers for the microbiome of healthy and lesion stages of DD have been well described and specific phyla representative of the various stages of disease have been identified. Firmicutes and Actinobacteria dominate healthy skin, Spirochetes dominate active stages of the disease and Firmicutes, Bacteroidetes and Proteobacteria dominate chronic or inactive stages [15–18]. Until recently, the transmission route and potential reservoir for the disease were unclear. Shotgun and 16S metagenomic analyses have now identified DD-associated Treponemes in rumen fluid, fecal and environmental samples [16,19]. This suggests the gut microbiota as the reservoir for microbes associated with DD pathogenesis and manure slurry as the primary transmission route [16,19].

Metagenomic analysis of 16S ribosomal RNA genes (rRNA) has been used to describe the microbial diversity of healthy and lesion stages of DD, however information on gene abundance and function is still unknown [15,16,19]. Shotgun metagenomic DNA sequencing has provided valuable insights into the phylogenetic composition, species diversity, metabolic capacity and functional diversity for a variety of biomes [20]. This technique has the potential to identify unknown etiological agents to better understand disease pathogenesis and to help devise preventative strategies as well as treatments against microbial disease agents.

Therefore, the objective of this study was to undertake a comparative evaluation of a microbial population and to understand composition and abundance of functional genes associated with healthy skin and diseased skin using metagenomic DNA shotgun sequencing, to obtain a greater comprehension of the etiopathogenesis of the disease.

## Materials and Methods

### Ethics statement

This study was carried out in strict accordance with the recommendations of The Animal Welfare Act of 1966 (AWA) (P.L. 89–544) and its amendments 1970 (P.L. 91–579); 1976 (P.L. 94–279), 1985 (P.L. 99–198) that regulates the transportation, purchase, care, and treatment of animals used in research. The research protocol was reviewed and approved by the Institutional Animal Care and use Committee of Cornell University (Protocol number: 2008–0096). The skin biopsy sample collections from cattle affected with DD were authorized by the farm owner, who was aware of the procedure.

### Healthy skin and digital dermatitis lesion sample collection

Samples from healthy skin and DD lesions were collected from Holstein dairy cows housed in one dairy farm located near Ithaca, NY (Latitude: 42.728595; Longitude: -76.571955). Lesion classification was based on the scoring method as described by Zinicola et al. (2015) [16]. Briefly, lesions with areas of ulceration were considered as active digital dermatitis (ADD), lesions characterized by the presence of firm scab, hyperkeratosis, proliferative overgrowth, and the absence of an ulcerative area were classified as inactive digital dermatitis (IDD). A total of 16 biopsy samples were collected from 8 dairy cows, 4 from ADD, 4 from IDD and 8 from healthy skin (HS), which were harvested using a 0.6 cm diameter punch biopsy instrument (Biopsy Punch, Miltex Inc., PA). The punch biopsy was performed in the center of the DD lesion. Healthy skin samples were collected from the same hoof affected by DD, 2 cm from the center of the DD lesions. For all biopsy samples, the biopsy area was cleaned and disinfected with clorhexidine (Clorhexidine 2%; VetOne, Boise, ID) and a local anesthesia was performed by 3 subcutaneous injections of 2 mL of lidocaine (Lidocaine 2%; VetOne, Boise, ID). Following collections, samples were placed in sterile 2.0 ml microcentrifuge tubes, transported on ice and stored at -80°C until further analysis.

### DNA extraction

HS, ADD and IDD samples were washed 3 times with nuclease-free water and incubated at 56°C for 12 h with 40 μl of proteinase K (IBI Scientific), 180 μl of tissue lysis buffer and 40 μl of lysozyme (QIAamp DNA Minikit, Qiagen, Valencia, CA, USA) to maximize bacterial DNA extraction. 250 mg of post-incubation healthy skin samples and DD lesions were placed in PowerBead Tubes (PowerSoil DNA Isolation kit, MO BIO Laboratories, Inc., Carlsbad, CA, USA) and settled in a Mini-Beadbeater-8 (Biospec Products, Battersville, OK, USA) for microbial cell disruption. DNA extraction was performed using a PowerSoil DNA Isolation Kit (MO BIO Laboratory Inc.) following the manufacturer’s recommendation. DNA concentration was evaluated using the Quant-iT PicoGreen dsDNA Assay Kit (Life Technologies Corporation, Carlsbad, CA, USA).

### Whole genome-Shotgun sequencing

An aliquot of each extracted sample was normalized to 0.2 ng/μl. Once the normalization was done the samples could be used as an input to the Nextera XT DNA Sample Prep Kit (Illumina Inc. San Diego, CA). Tagmentation of samples was done using 1 ng of template, as directed by manufacturer. Following tagmentation, PCR amplification was done according to manufacturer’s instructions using a unique combination of barcode primers (provided by manufacture) for each of the 16 samples allowing samples to be multiplexed. Following amplification, each DNA library had short DNA fragments removed using AMPure XP bead purification and then normalized through Library Normalization beads/additives. In addition, to prepare for cluster generation and sequencing, equal volumes of normalized libraries were combined, diluted in hybridization buffer and heat denatured, according to Nextera XT protocol. Finally, Pair-end sequencing was performed using the MiSeq Reagent Kit v3 (600-cycles) through the Illumina MiSeq platform.

### Bioinformatics and statistical analysis

Raw data files from shotgun sequencing were de-multiplexed and converted to fastq using Casava v.1.8.2 (Illumina, Inc, San Diego, CA, USA). Fastq files were concatenated and uploaded to the MG-RAST [21] server for further analysis, using two annotation source data, SEED and M5NR. In MG-RAST, sequences were subjected to quality control, which includes dereplication (remove artificial sequences produced by sequencing artifacts), removing host specific species sequences (B. Taurus, UMD v3.0), ambiguous base filtering (removing sequences with >5 ambiguous base pairs) and a length filtering (removing sequences with a length of >2 standard deviation from the mean). Organism abundance was analyzed using a “Best Hit Classification” approach with a maximum e-value of 1 x 10^−5^, minimum identity of 60% and a minimum alignment length of 15 measured in amino acids for proteins and base pairs for RNA databases. A functional abundance analysis was performed using “Hierarchical Classification”, with a maximum e-value of 1 x 10^−5^, minimum identity of 60% and a minimum alignment length of 15 measured in amino acids for proteins and base pairs for RNA databases. One sample with less than 1,000 sequences from IDD was excluded, yielding 15 samples for downstream analyses. To reduce the impact of experimental noise/error, the normalized data option of MG- RAST was used. Data underwent a normalization procedure, all values have undergone a log2-based transformation (log2 (x + 1)) followed by standardization within each sample and linear scaling (across all samples). Details can be found in the MG-RAST manual (ftp://ftp.metagenomics.anl.gov/data/manual/mg-rast-manual.pdf).

Phyla taxa abundance, normalized abundance of *Treponema* spp., flagellar motility, flagellum proteins, bacterial chemotaxis, genus distribution (unclassified, derived from Eukaryota), *Candidatus Aemobophilus asiaticus* and distribution of resistance to antibiotics and toxic compounds from HS, ADD and IDD were extracted from MG-RAST and analyzed using ANOVA in conjunction with Tukey test for multiple comparisons using JMP Pro 11(SAS Institute Inc., NC). A series of multivariable screening analyses using JMP Pro 11 were performed to determine which flagella protein were most important in differentiating the HS microbiome from the microbiomes of ADD and IDD. The false discovery rate (FDR) [22] was used to correct for multiple comparisons. Heatmap for phyla and function distribution from HS, ADD and IDD, and circular tree of *Treponema* strains from ADD and IDD lesion states were performed using MG-RAST. Results are presented as least square means follow by the standard error of the mean unless stated otherwise.

Several recruitment plots for each sample of HS (4567753.3, 4567754.3, 4567755.3, 4567756.3, 4567757.3, 4567758.3, 4567759.3 and 4567760.3), ADD (4567749.3, 4567750.3, 4567751.3 and 4567752.3) and IDD (4567761.3, 4567762.3, and 4567763.3) were mapped against the genomes of *T*. *denticola* ATCC 35405, *T*. *vincentii* ATCC 35580 and *T*.*pallidum* subsp. *pallidum str*. *Nichols* using MG-RAST.

## Results

### Sequencing data

Whole-genome sequencing was employed to compare HS, ADD and IDD from 16 dairy cows affected by DD. Table 1 illustrates the sequencing data obtained.

**Table 1:** Summary of sequencing data.

Sample Identification^1^	Lesion Stage	Total Sequences (bp)	Sequences Read	Sequences length (bp)
4567753.3	Healthy	243.696.864	845.264	288 ± 100
4567754.3	Healthy	232.588.313	796.843	291 ± 101
4567755.3	Healthy	237.286.216	849.513	265 ± 101
4567756.3	Healthy	248.573.941	931.149	266 ± 100
4567757.3	Healthy	221.572.287	729.257	303 ± 104
4567758.3	Healthy	201.191.574	643.647	312 ± 104
4567759.3	Healthy	213.142.025	658.555	323 ± 102
4567760.3	Healthy	126.802.521	381.654	332 ± 107
4567749.3	Active	80.612.233	326.895	246 ± 97
4567750.3	Active	144.507.806	486.126	297 ± 106
4567751.3	Active	112.388.248	382.614	293 ± 98
4567752.3	Active	222.540.933	781.919	284 ± 103
4567761.3	Inactive	139.721.802	467.74	298 ± 105
4567762.3	Inactive	141.160.009	483.136	292 ± 101
4567763.3	Inactive	158.419.942	642.364	246 ± 105

^1^Sequences have been deposited in the MG-RAST database (http://metagenomics.anl.gov/metagenomics.cgi?page=Home) and can be found under these sample identification codes.

### Normalized abundances

**Fig 1:**
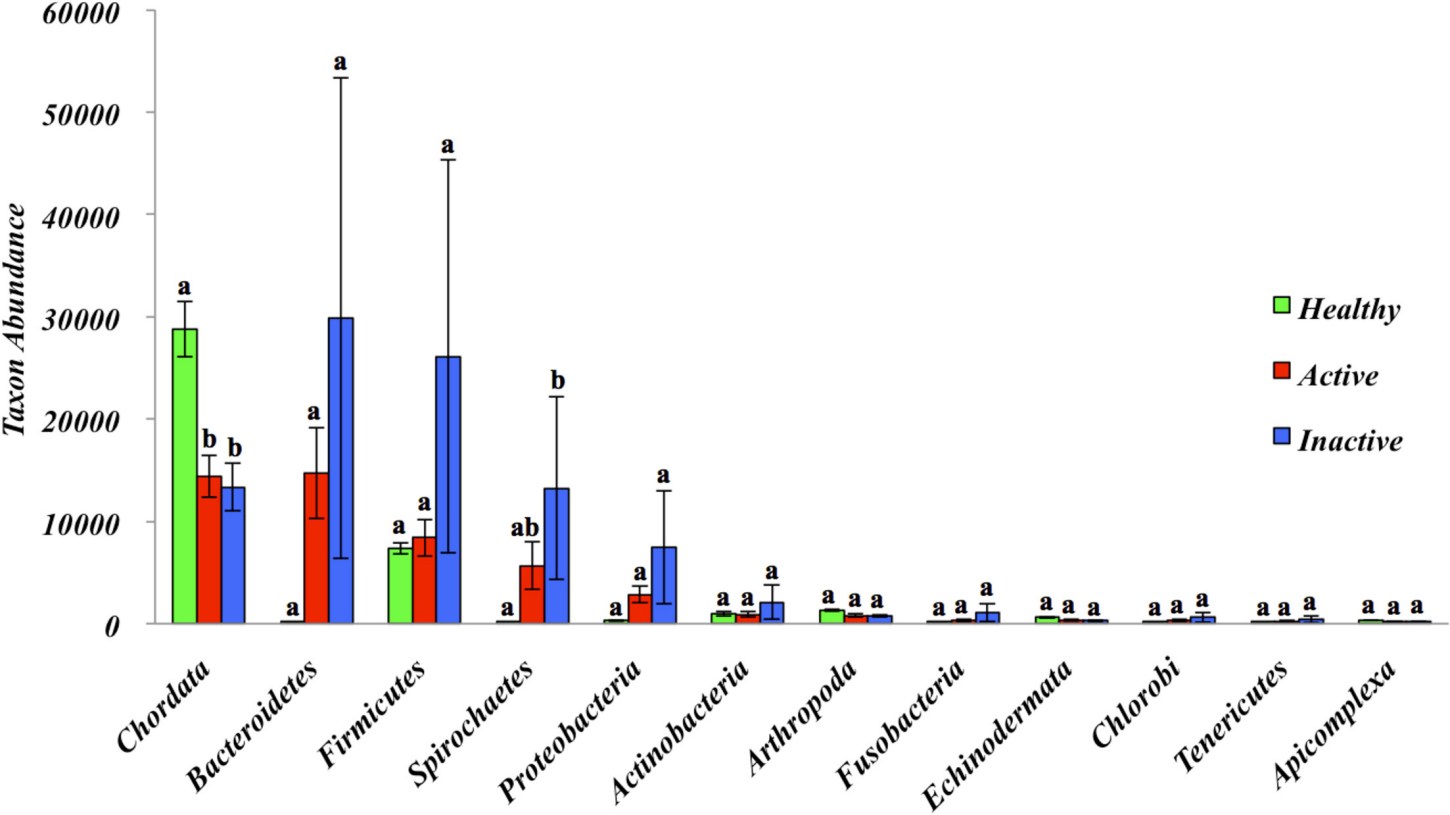
Phyla abundances ordered from the most abundant to the least abundant by HS, ADD and IDD. Only the top 12 most abundant phyla are shown. The y-axis represents the mean abundances of annotation in each phylum on a log scale. Error bars are standard error of the mean. Different letters means *P* < 0.05.

**Fig 2:**
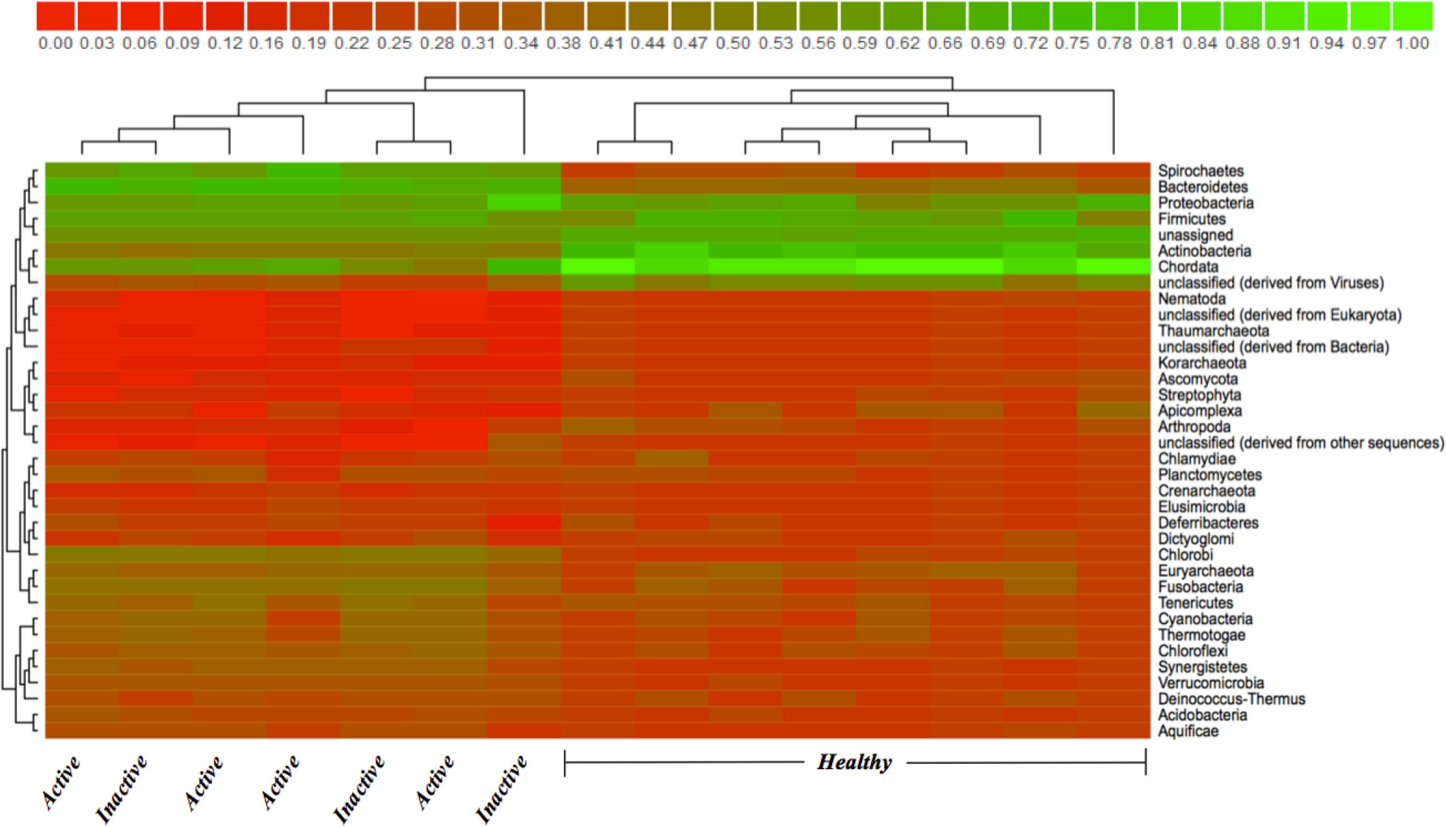
MG-RAST heatmap depicting phyla distribution from ADD, IDD and HS. Red and green colors scale represents low and high abundance, respectively.

**Fig 3:**
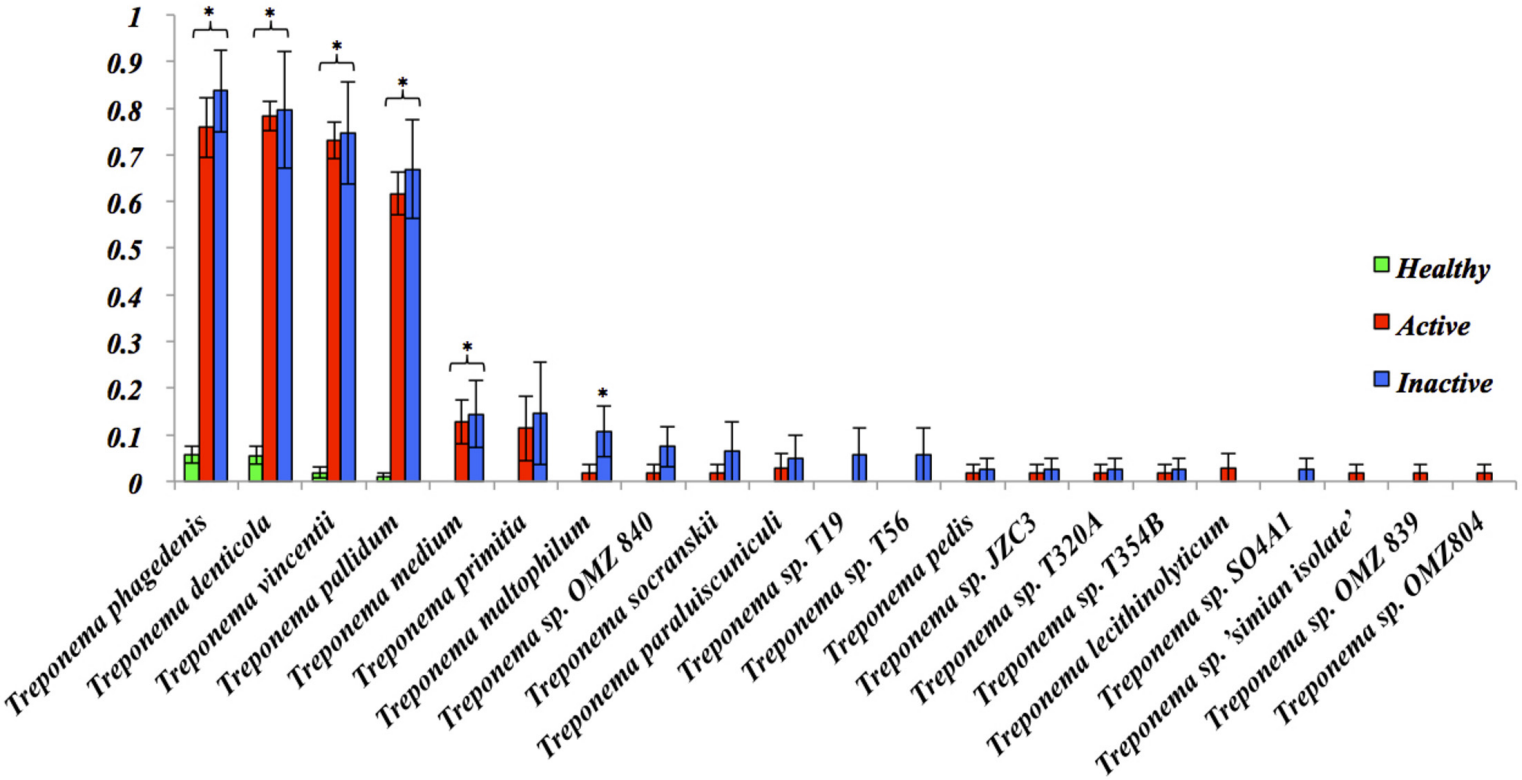
Normalized abundance of *Treponema* spp. from HS, ADD and IDD. Error bars are standard error of the mean. *P < 0.05.

**Fig 4:**
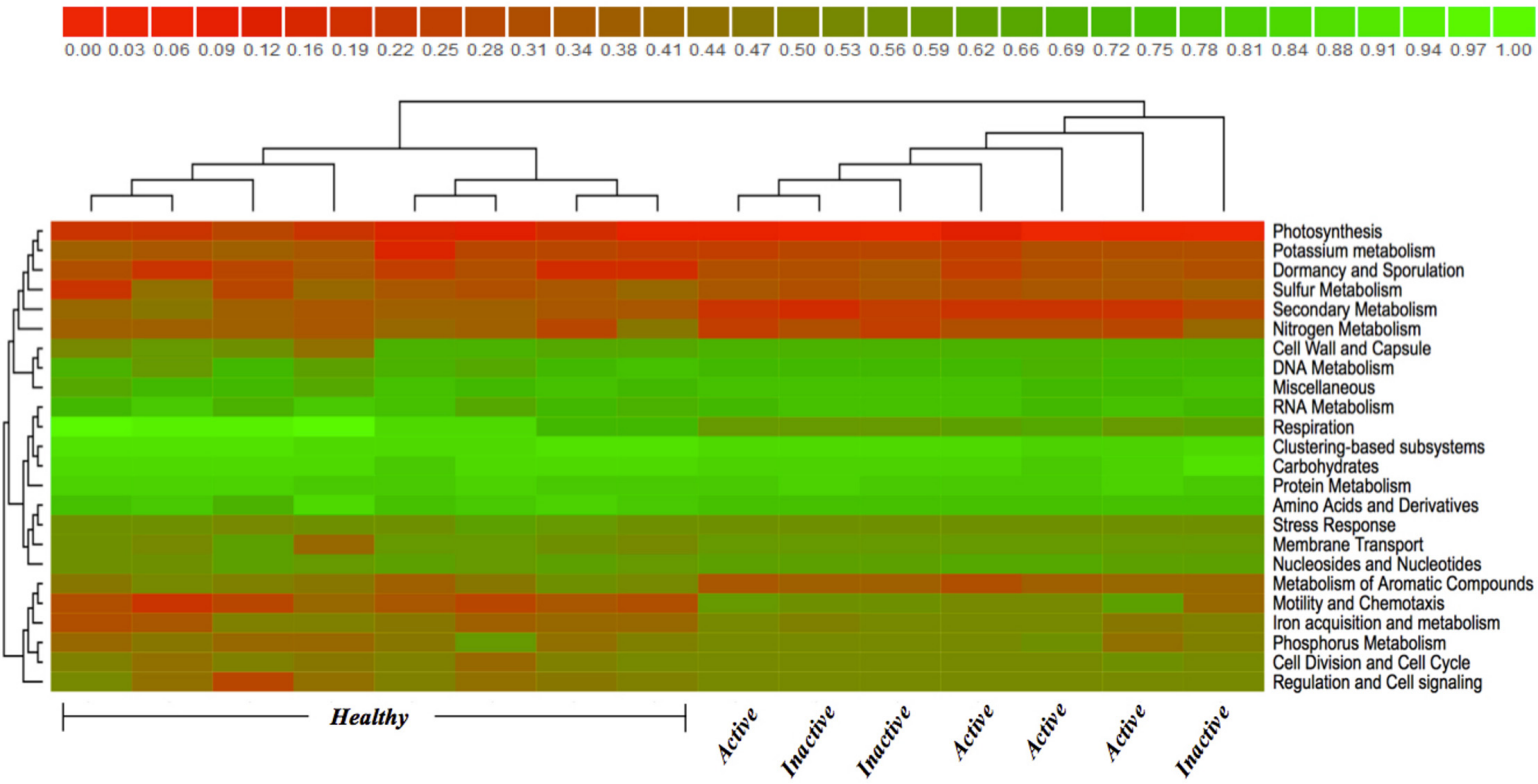
MG-RAST heatmap displaying function distribution from ADD, IDD and HS. Red and green colors scale represents low and high abundance, respectively.

Comparison of the 12 most abundant phyla between HS, ADD and IDD are illustrated in Fig 1. *Chordata*, *Firmicutes*, *Actinobacteria*, *Arthropoda*, and *Echinodermata* were identified in HS as the most abundant phyla with *Chordata* being the most dominant phylum. Statistically significant differences at the phylum level were not identified between ADD and IDD and *Chordata*, *Bacteroidetes*, *Firmicutes*, *Spirochaetes and Protobacteria* were the most abundant phyla. The phylum *Chordata*, most likely bovine DNA, was more abundant (*P*<0.05) in HS when compared to ADD and IDD and the phylum *Spirochaetes* was significantly more abundant (*P*<0.05) in IDD when compared to HS (Fig 1). We also evaluated the phylum distribution of HS, ADD and IDD samples using a Heatmap (Fig 2). *Spirochaetes* and *Bacteroidetes* were highly prevalent in ADD and IDD samples followed by *Proteobacteria* and *Firmicutes* (Fig 2). The most dominant phyla in HS samples include *Chordata*, *Actinobacteria* and *Firmicutes* (Fig 2). Analysis of the normalized abundance of *Treponema* spp. from HS, ADD and IDD demonstrated that *T*. *phagedenis*, *T*. *denticola*, *T*. *vincentii* and *T*. *pallidum* were the most abundant *Treponema* spp. and were significantly more abundant (*P*<0.05) in ADD and IDD compared to HS samples (Fig 3). Evaluation of the *Treponema* spp. across ADD and IDD identified a higher abundance (*P*<0.05) of *T*. *maltophilum* in IDD compared to ADD (Fig 3). The heatmap showing the functional composition of HS, ADD and IDD samples is illustrated in Fig 4. Differences in functional categories were associated with motility/chemotaxis, respiration, iron acquisition, phosphorus metabolism, cell division and cell cycle and regulation and cell signaling (Fig 4).

**Fig 5:**
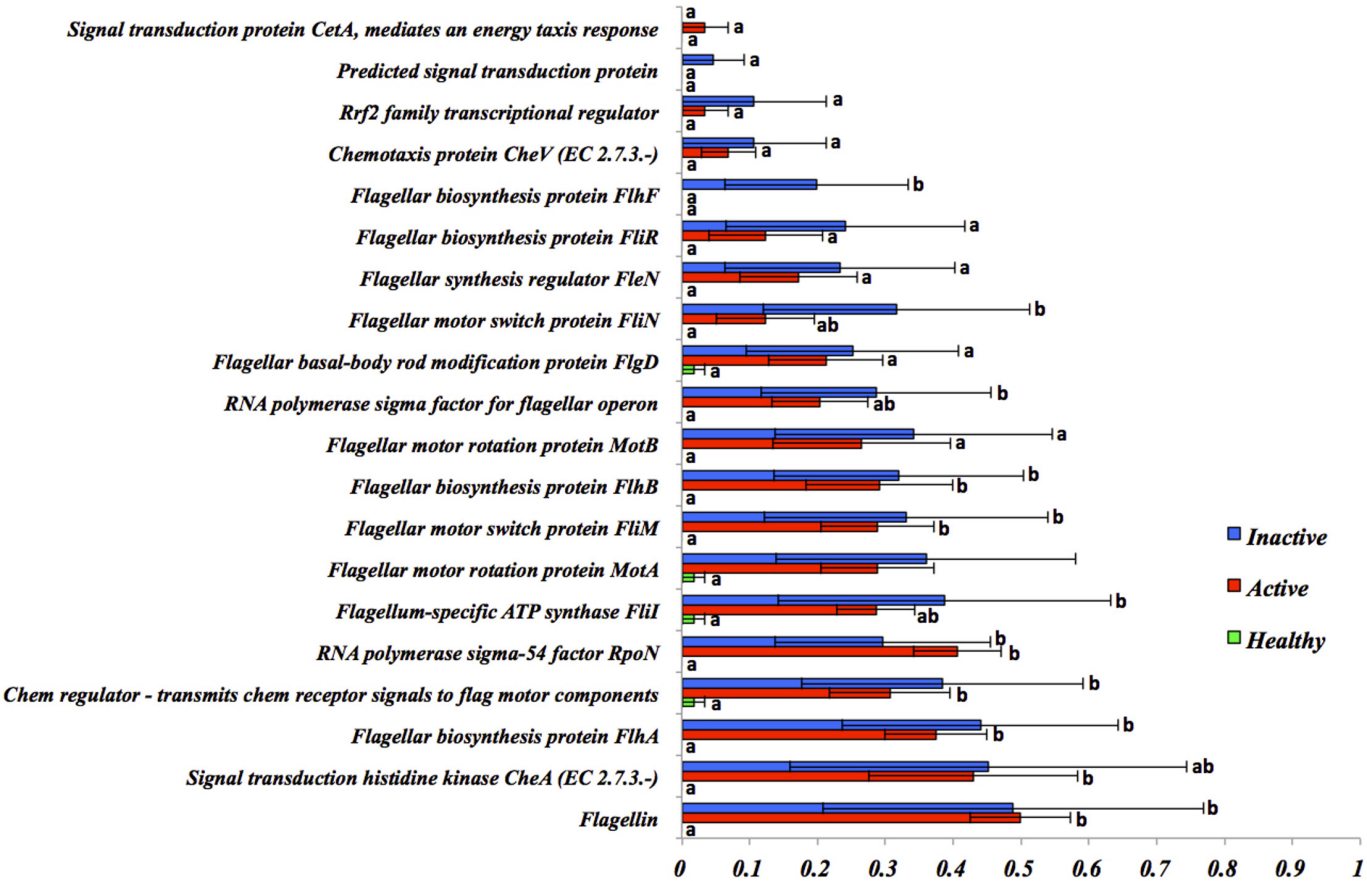
Normalized abundance of genes related with flagellar motility in HS, ADD and IDD. Error bars are standard error of the mean. Different letters means *P* < 0.05.

**Fig 6:**
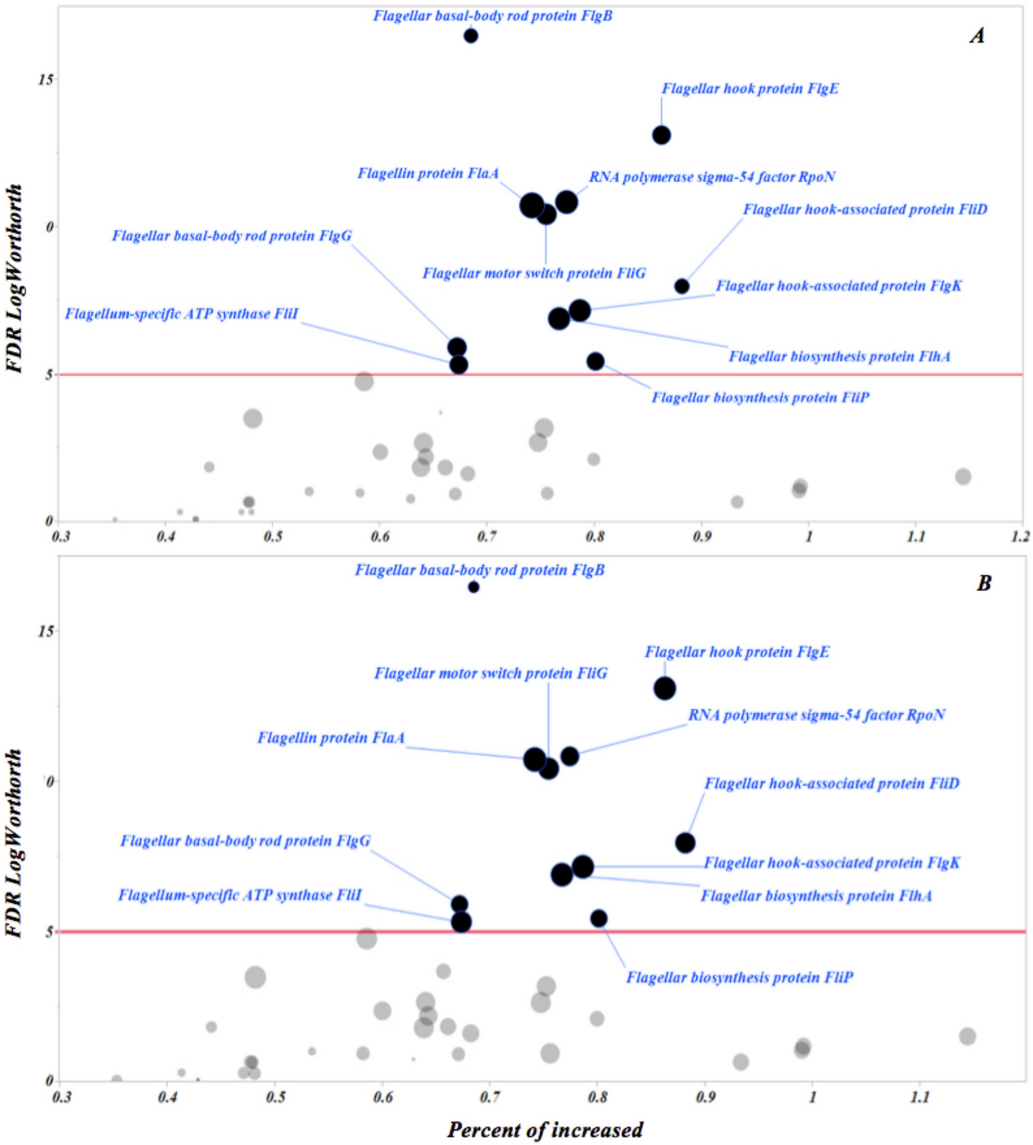
Bubble plot representing differences in abundance of flagellum associated proteins between lesions and healthy skin (Fig 7A: active lesion vs. healthy skin and Fig 7B: inactive lesion vs. healthy skin). The Y axis represents the robust LogWorth of the false discovery rate and the X axis represents the percentage of prevalence when comparing healthy skin to lesions stages. The size of the circles denotes the effect size. Red line represents *P* < 0.00005.

To assess putative virulence factors associated with *Treponema* spp. and DD, the normalized abundance of genes associated with flagellar motility, bacterial chemotaxis and flagellar proteins from HS, ADD and IDD was evaluated and is illustrated in Figs 5 and S1 and S1 Table, respectively. The majority of genes with a role in flagella structure and synthesis in *Treponema* spp. were overrepresented in ADD and IDD when compared to HS. Two screening analyses were performed to identify the most prevalent and dominant flagella proteins for HS to ADD and IDD samples. The results indicated that 11 genes were distinct (*P*<0.00005) and highly prevalent in ADD and IDD compared with HS (Fig 6).

**Fig 7:**
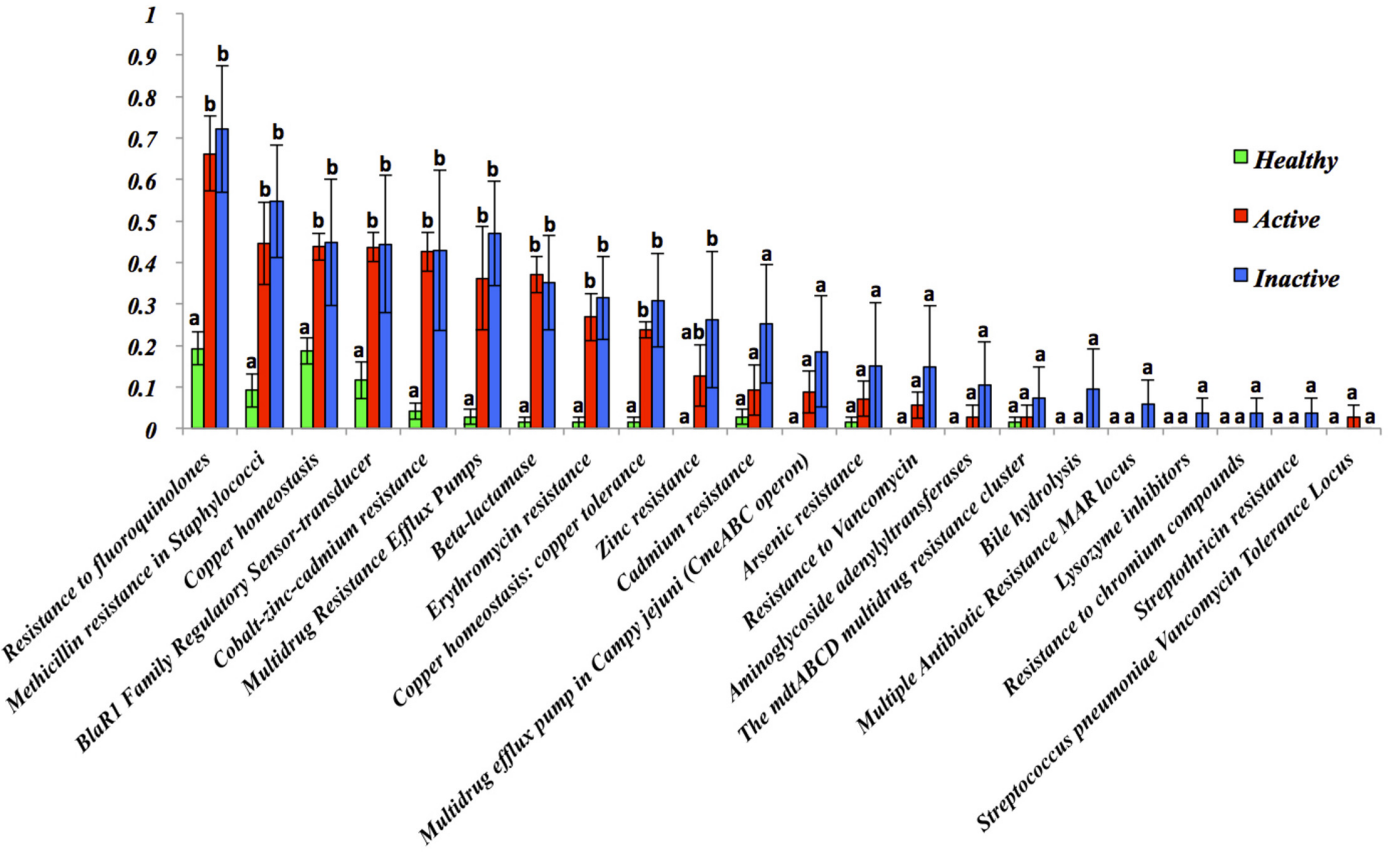
Normalized abundance distribution of resistance to antibiotics and toxic compounds of HS, ADD and IDD. Error bars represent standard error of the mean. Different letters means *P* < 0.05.

Comparison of normalized abundance of distribution of resistance to antibiotics and toxic compounds for HS, ADD and IDD is presented in Fig 7. It is evident that ADD and IDD have increased antibiotic resistance compared to that of healthy samples. Comparison of the antibiotic resistance profiles for IDD and ADD shows a similar trend for both samples.

The normalized abundance of genus distribution (unclassified, derived from Eukaryota) was compared for HS, ADD and IDD. The data indicates the presence of *Acanthamoebas* (free-living amoebas) in ADD and IDD (S2 Fig). Comparison of the normalized abundance of *Candidatus Aemobophilus asiaticus* demonstrated the presence of this bacterium in ADD (0.35 ± 0.12) and IDD (0.44 ± 0.14) but the bacterium was completely absent in HS.

### Recruitment plots and Circular tree

**Fig 8:**
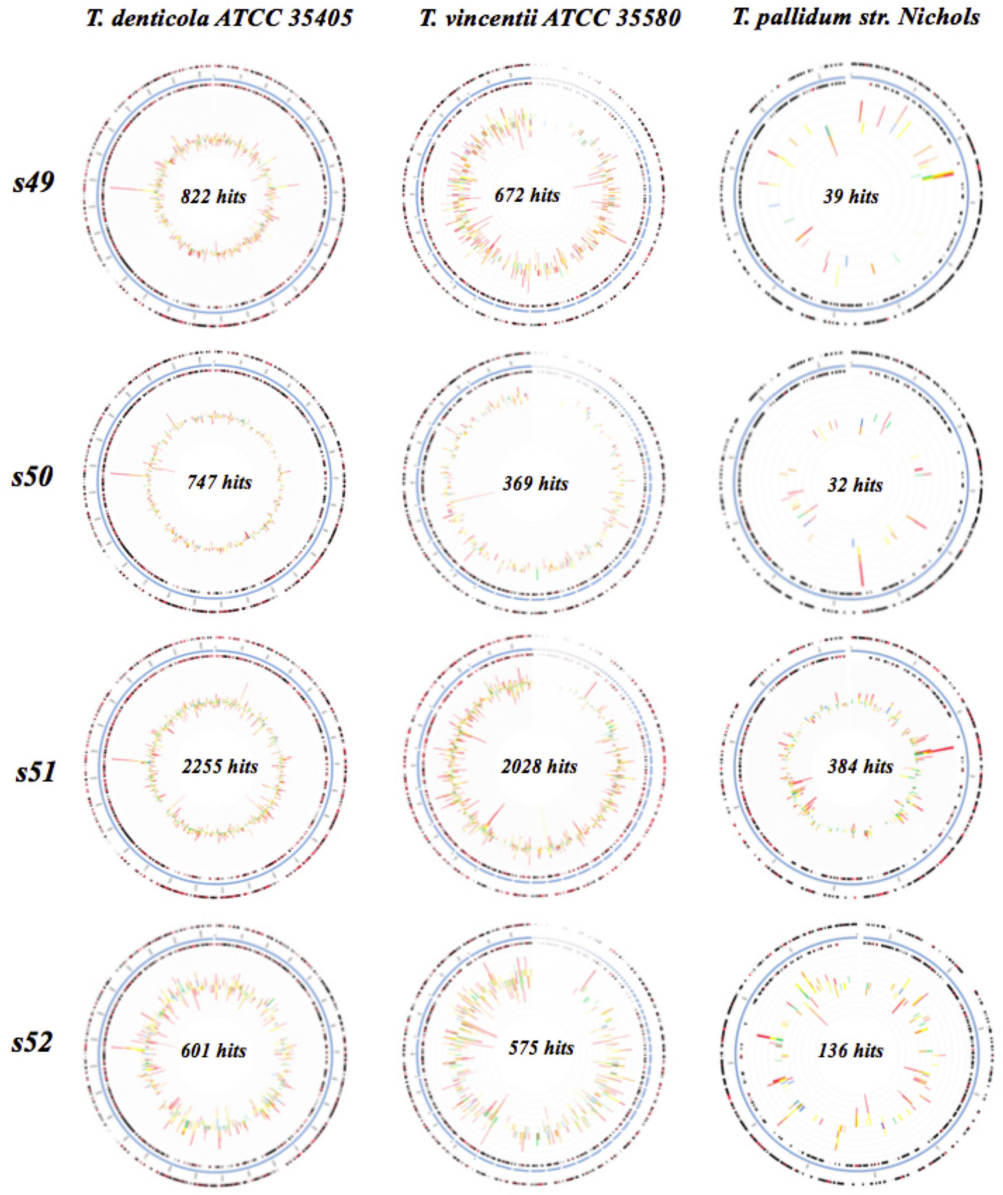
Recruitment plot for ADD (samples 4567749.3, 4567750.3, 4567751.3, 4567752.3) mapped on *T*. *denticola* ATCC 35405, *T*. *vincentii* ATCC 35580 and *T*.*pallidum* subsp. *pallidum str*. *Nichols*. The blue circle represents the bacterial contigs for the genome of interest, while the two black rings map genes on the forward and reverse strands. Bars represent hits distributions and the colors are coded according to the e-value of the matches with red (-30 and less), orange (-20 to -30), yellow (-10 to -20), green (-5 to -10) and blue (-3 to -5).

**Fig 9:**
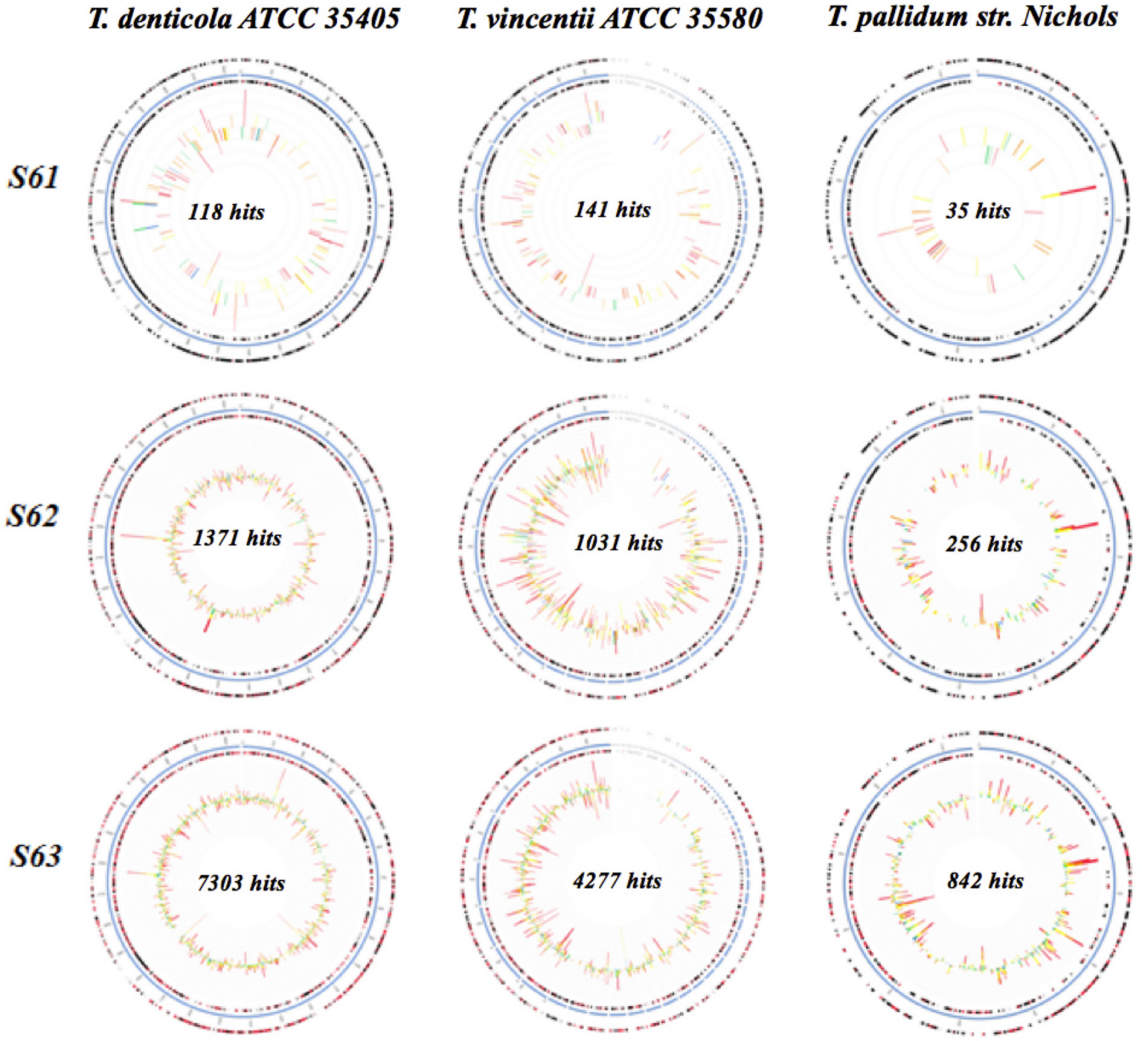
Recruitment plot for IDD (samples 4567761.3, 4567762.3 and 4567763.3) mapped on *T*. *denticola* ATCC 35405, *T*. *vincentii* ATCC 35580 and *T*.*pallidum* subsp. *pallidum str*. *Nichols*. The blue circle represents the bacterial contigs for the genome of interest, while the two black rings map genes on the forward and reverse strands. Bars represent hits distributions and the colors are coded according to the e-value of the matches with red (-30 and less), orange (-20 to -30), yellow (-10 to -20), green (-5 to -10) and blue (-3 to -5).

Recruitment plots comparing sequences from each set of HS, ADD and IDD samples to the specific genomes of *T*. *denticola* ATCC 35405, *T*. *vincentii* ATCC 35580 and *T*. *pallidum* subsp. *pallidum str*. *Nichols* confirmed the homology of the sample sequences to each specific genome (Figs 8 and 9). The analysis revealed a greater number of sequences with identity to features of *T*. *denticola* ATCC 35405 and *T*. *vincentii* ATCC 35580 genomes in both ADD and IDD samples. Comparison of *T*. *pallidum* subsp. *pallidum str*. *Nichols* genome to ADD and IDD sequences was inconsistent. For HS samples a limited number of hits matched to the genome of the *Treponema* spp. (S3 Fig). Hits were not identified for samples 4567751.3, 4567752.3, 4567754.3, 4567755.3, 4567757.3, 4567759.3 and 4567760.3 (S3 Fig). Circular tree of ADD and IDD revealed a high number of sequence reads associated with *T*. *denticola* ATCC 35405, *T*. *vincentii* ATCC 35580, *T*.*pallidum* subsp. *Pallidum*, *T*. *phagedenis* F042 and *T*. *phagedenis* (S4 Fig). Other *Trepoema* spp. with relatively low numbers of sequences were identified (S4 Fig).

## Discussion

Metagenomic methods amplifying 16S ribosomal RNA genes (rRNA) have been used to describe the bacterial and archaeal phylogenetic composition of healthy and lesion stages of DD [15,16,19]. However, there is a lack of information regarding the functional composition of the metagenome associated with the disease. Whole genome shotgun sequencing of DNA is another approach to study microbial composition and diversity. It has previously been demonstrated that while data generated from 16S rRNA and shotgun sequencing are distinct [23,24], there is a huge overlap. Moreover, PCR bias is largely reduced in whole genome sequencing. The present study used shotgun metagenomics and the MiSeq Illumina sequencing platform to describe the microbial diversity. MG-RAST metagenomic analysis was used to assess gene function in HBS, ADD and IDD of dairy cows affected by DD.

*Bacteroidetes*, *Firmicutes*, *Spirochaetes*, *Proteobacteria* and *Actinobacteria* were the most abundant bacterial phyla across all three samples types (HS, ADD, and IDD). The most abundant bacterial phyla identified in ADD and IDD samples were *Spirochetes* followed by *Bacteroidetes*, *Firmicutes* and *Proteobacteria*, which is in agreement with previous studies that have identified these phyla as the most abundant in DD lesions [15,16,19]. The two most abundant bacterial phyla in HS samples were *Firmicutes* and *Actinobacteria*, which is in line with data generated from other studies, identifying these three phyla as the most common phyla in healthy samples [15–17]. From the data obtained in our study it can be concluded that there is a distinct difference be tween the phyla types associated with DD samples compared to that of healthy samples.

DD was first perceived to be a polymicrobial disease, however more comprehensive studies have identified *Treponema* spp. as key components in the microbial etiology of the disease [18,19,25]. In our work we identified specific species of *Treponema* namely *T*. *denticola-like*, *T*. *phagedenis-like* and *T*. *vincentii-like* as overrepresented in ADD and IDD compared to HS. This finding is consistent with work conducted by Evans et al. (2008) [25], where they identified *T*. *phagedenis-like*, *T*. *medium/T*. *vincentii-like* and *T*. *putidum/T*. *denticola-like* in bovine DD samples in abundances of 98%, 96.1% and 74.5%, respectively [25]. In the present study, we performed recruitment plot analyses where metagenomic sequences from HS, ADD and IDD samples were aligned against the genomes of *T*. *denticola* ATCC 35405, *T*. *vincentii* ATCC 35580 and *T*. *pallidum* subsp. *pallidum str*. *Nichols*. These recruitment plots determined that ADD and IDD sequences mapped with a high degree of similarity to *T*. *denticola* ATCC 35405 and *T*. *vincentii* ATCC 35580, signifying the presence of these specific *Treponema* spp. in diseased samples. Conversely, we established that a low number of sequences mapped to *T*. *pallidum* subsp. *pallidum str*. *Nichols* suggesting that our *Treponema* isolate does not belong to *T*. *pallidum* subsp. *pallidum str*. *Nichols*. Recruitment plot analysis was not performed for *T*. *phagedenis* and *T*. *medium* because their complete genomes were not fully sequenced at the time of data analysis. Circular tree analysis of the *Treponema* strains associated with ADD and IDD identified a high number of sequence reads for these specific *Treponema* spp. A small number of sequence reads were observed for other *Treponema* spp. previously associated with DD, including *T*. *maltophilum*, *T*. *medium*, *T*. *pedis* and *T*. *paruluiscuniculi* [16–18,25]. However, this low association may potentially be due to the small sample size used in this study.

Differences in functional features across the microbiomes of HS, ADD and IDD were identified through our MG-RAST analyses. We predominantly focused on further evaluating the most significantly altered functional features. Treponemas are characterized as Gram-negative, motile, anaerobic bacteria and are a member of the *Spirochetes* phylum. Typically *Spirochetes* have a unique structure consisting of a periplasmic flagellum located between the inner and the outer membrane, thus distinguishing these from other bacteria. Depending on the extracellular environmental stimuli, chemotaxis enables *Spirochetes* to move towards specific niches for colonization [26,27]. The ability to penetrate viscous media such as the skin tissue is an important virulence factor associated with pathogenic *Spirochetes* [28–30]. Lux et al. (2001) [31] established an *in vitro* tissue penetration assay using wild-type *T*. *denticola*, *T*. *pallidum* and *T*. *phagedenis* and demonstrated the ability of *T*. *denticola* and *T*. *pallidum* to penetrate tissue. However *T*. *phagedenis* was unable to overcome the epithelial barrier, suggesting that during the penetration process some *Treponema* spp. facilitate the entry of other Treponemes [31]. An association between *Treponema* spp. and deeper layers of skin affected by DD has been identified, thus further supporting the ability of *Treponema* spp. to penetrate skin tissue [16,18].

To gain a deeper insight into the virulence factors employed by DD-associated *Treponema* spp., comparison of the functional composition of HS compared to ADD and IDD was completed. Motility and chemotaxis were determined to be dominant functional features. A large portion of genes identified in ADD and IDD were related to flagella structure and to genes previously identified as playing a role in flagella synthesis in *Treponem*a spp. Screening analysis to detect highly prevalent flagella genes in HS, ADD and IDD identified eleven genes, which did not exhibit a distinct difference between ADD and IDD. Although the function of FlaA sheath flagella protein is not well understood, it has been suggested that this protein increases rigidity of the flagellum and may permit more efficient swimming in viscous environments like the skin tissue [32–34]. Our results showed a high prevalence of FlaA sheath protein in ADD and IDD compared to HS samples.

Chemotaxis signals enable bacteria to move directly in response to environmental stimuli through a network of complex signal transduction pathways [26,35]. Sim et al., (2005) [35] constructed a model of *T*. *denticola* chemotaxis signaling pathways and demonstrated the role of specific proteins involved in chemotaxis signaling, specifically the role of CheA, CheW, CheY, Che X, Che R and chemoreceptors [35]. When the normalized abundance of bacterial chemotaxis genes in HS, ADD and IDD was analyzed, all genes were highly present in ADD and IDD compared to HS. Our findings strongly support a role for motility and chemotaxis in the disease process of DD. Another significant functional feature detected in ADD and IDD was iron acquisition and metabolism. Iron is a crucial component for growth and development of almost all bacteria [36–39]. Vertebrates are devoid of free iron; subsequently pathogenic bacteria employ multiple systems to sense and sequester iron in an iron-limited milieu and transport this across the outer membrane into the cell [40]. Our results indicate that during infection, *Treponema* spp. express genes possibly related to surface receptors. A putative role for these genes would be in iron acquisition, however further work to characterize these is required.

Foot bathing using disinfectant or antibiotic solution has been the most common method routinely used to prevent and control DD. However, there is controversy regarding foot bathing strategies and their success. Speijers et al. (2010) [41] compared the efficacy of several foot bathing methods and solutions in the treatment of DD. They demonstrated that 5% of CuSO4 per week was the optimum treatment [41]. The relative efficacy of a commercial disinfectant agent used twice a week compared to using 5% formalin and 10% CuSO4 was assessed and it was established that the commercial product had greater overall efficacy [10]. However, Laven and Hunt (2002) [8] reported that hoof bathing daily for seven days with 5% formalin and 10% CuSO4 was effective in controlling DD [8]. Although it appears that CuSO4 is effective, most published studies do not include a negative control group [42]. Here we have identified a higher normalized abundance in the expression of genes associated with resistance to antibiotic and toxic compounds in ADD and IDD, namely copper and zinc. The chronicity of this disease has been explained as a consequence of unsuccessful immune response and the ability of Treponemes to change from spiral to encysted form in deeper layers of the skin [38,43–46]. This resistance may represent a mechanism by which *Treponema* spp. can persist in lesions. However, further research is needed to demonstrate the efficacy of CuSO4. Moreover, resistance was not observed against antibiotics such as oxitetracycline and lincomycin HCl, which are commonly used to topically treat DD [47].

A novel bacterium *Candiatus Aemobophilus asiatiaticus* has been identified in DD lesion samples and it is thought to play an important role in the pathogenesis of the disease [16]. It has also been reported that this bacterium has a symbiotic relationship with amoebas [48,49] and thus a second possibility is that *Candiatus Aemobophilus asiatiaticus* is an indirect predictor of amoebas [16]. Although in the present study we demonstrated the presence of this bacterium in ADD and IDD, we confirm the presence of *Acanthamoebas* in ADD and IDD supporting the hypothesis that *Candiatus Aemobophilus asiatiaticus* indirectly indicates the existence of free-living amoebas without necessarily being involved in DD pathogenesis.

Amplicon sequencing of highly conserved genes, such as segments of the 16S rRNA gene, have been used in order to determine which organisms were present in different stages of DD lesions [15,16,18,19]. These studies, using a large sample size, identified differences among the microbiome of ADD and IDD. However, the present study did not identify differences among the microbiome of ADD and IDD and are likely attributed to the restricted number of samples and the experimental method used. Despite these differences, the technique used in this study revealed the potential metabolic profiles of a microbial community. How this potential is translated to functional activity remains unknown and further work needs to be completed to identify which genes are differentially expressed in ADD and IDD measured by metatranscriptome.

## Conclusion

Here, we report the first shotgun metagenomic study of the microbiome of bovine digital dermatitis lesions. The MiSeq Illumina sequencing platform was used to assess the microbial diversity of DD and *T*. *denticola-like*, *T*. *phagedenis-like* and *T*. *vincentii-like* were identified as the dominant strains present in ADD and IDD. MG-RAST metagenomic analysis identified virulence factors including motility/chemotaxis and iron uptake. Additionally, an increase in the abundance of copper and zinc resistance genes has been reported, suggesting that further research is necessary to optimize the foot bathing technique for the control and treatment of DD. Overall, our findings provide significant information to better understand disease pathogenesis as well as to settle preventative and efficient treatments against microbes involved in DD pathogenesis.

## Supporting Information

S1 Fig Normalized abundance of bacterial chemotaxis in HS, ADD and IDD. Error bars represented the standard error of the mean. Different letters means *P* < 0.05.
(TIF)

S2 Fig Normalized abundance of genus distribution (unclassified, derived from Eukaryota) in HS, ADD and IDD. Error bars represent standard error of the mean.
(TIF)

S3 Fig Recruitment plot for healthy samples (4567753.3, 4567756.3 and 4567758.3) mapped on *T*. *denticola* ATCC 35405, *T*. *vincentii* ATCC 35580 and *T*.*pallidum* subsp. *pallidum str*. *Nichols*. The blue circle represents the bacterial contigs for the genome of interest, while the two black rings map genes on the forward and reverse strands. Bars represent hits distributions and the colors are coded according to the e-value of the matches with red (-30 and less), orange (-20 to -30), yellow (-10 to -20), green (-5 to -10) and blue (-3 to -5).
(TIF)

S4 Fig Circular tree of *Treponema* starins of active and inactive stages. Each bar plot is related to the number of sequencing reads detected per bacterial association.
(TIF)

S1 Table Summary of normalized abundance of flagellum proteins of HS, ADD, and IDD. (PDF)

## References

[1] Holzhauer M, Hardenberg C, Bartels CJ, Frankena K. Herd- and cow-level prevalence of digital dermatitis in the Netherlands and associated risk factors. J Dairy Sci. 2006;89: 580–588. 16428627 10.3168/jds.S0022-0302(06)72121-X

[2] USDA. Dairy 2007, Part IV: Reference of Dairy Cattle Health and Management Practices in the United States. 2009;USDA:APHIS:VS, CEAH, Fort Collins, CO.

[3] Bruijnis MR, Beerda B, Hogeveen H, Stassen EN. Assessing the welfare impact of foot disorders in dairy cattle by a modeling approach. Animal. 2012;6: 962–970. 10.1017/S1751731111002606 22558967

[4] Garbarino EJ, Hernandez JA, Shearer JK, Risco CA, Thatcher WW. Effect of lameness on ovarian activity in postpartum holstein cows. J Dairy Sci. 2004;87: 4123–4131. 15545374 10.3168/jds.S0022-0302(04)73555-9

[5] Losinger WC. Economic impacts of reduced milk production associated with papillomatous digital dermatitis in dairy cows in the USA. J Dairy Res. 2006;73: 244–256. 16569275 10.1017/S0022029906001798

[6] Ettema J, Ostergaard S, Kristensen AR. Modelling the economic impact of three lameness causing diseases using herd and cow level evidence. Prev Vet Med. 2010;95: 64–73. 10.1016/j.prevetmed.2010.03.001 20371126

[7] Cha E, Hertl JA, Bar D, Grohn YT. The cost of different types of lameness in dairy cows calculated by dynamic programming. Prev Vet Med. 2010;97: 1–8. 10.1016/j.prevetmed.2010.07.011 20801533

[8] Laven RA, Hunt H. Evaluation of copper sulphate, formalin and peracetic acid in footbaths for the treatment of digital dermatitis in cattle. Vet Rec. 2002;151: 144–146. 12199433 10.1136/vr.151.5.144

[9] Holzhauer M, Dopfer D, de Boer J, van Schaik G. Effects of different intervention strategies on the incidence of papillomatous digital dermatitis in dairy cows. Vet Rec. 2008;162: 41–46. 18192655 10.1136/vr.162.2.41

[10] Teixeira AG, Machado VS, Caixeta LS, Pereira RV, Bicalho RC. Efficacy of formalin, copper sulfate, and a commercial footbath product in the control of digital dermatitis. J Dairy Sci. 2010;93: 3628–3634. 10.3168/jds.2010-3246 20655432

[11] Blowey RW, Carter SD, White AG, Barnes A. Borrelia burgdorferi infections in UK cattle: a possible association with digital dermatitis. Vet Rec. 1994;135: 577–578. 7886898

[12] Döpfer D, Koopmans A, Meijer FA, Szakall I, Schukken YH, Klee W, et al Histological and bacteriological evaluation of digital dermatitis in cattle, with special reference to spirochaetes and Campylobacter faecalis. Vet Rec. 1997;140: 620–623. 9228692 10.1136/vr.140.24.620

[13] Schlafer S, Nordhoff M, Wyss C, Strub S, Hubner J, Gescher DM, et al Involvement of Guggenheimella bovis in digital dermatitis lesions of dairy cows. Vet Microbiol. 2008;128: 118–125. 18024006 10.1016/j.vetmic.2007.09.024

[14] Rasmussen M, Capion N, Klitgaard K, Rogdo T, Fjeldaas T, Boye M, et al Bovine digital dermatitis: possible pathogenic consortium consisting of Dichelobacter nodosus and multiple Treponema species. Vet Microbiol. 2012;160: 151–161. 10.1016/j.vetmic.2012.05.018 22698300

[15] Krull AC, Shearer JK, Gorden PJ, Cooper VL, Phillips GJ, Plummer PJ. Deep Sequencing Analysis Reveals the Temporal Microbiota Changes Associated with the Development of Bovine Digital Dermatitis. Infect Immun. 2014. 10.1128/IAI.02077-14 PMC4136199 24866801

[16] Zinicola M, Lima F, Lima S, Machado V, Gomez M, Dopfer D, et al Altered Microbiomes in Bovine Digital Dermatitis Lesions, and the Gut as a Pathogen Reservoir. PLoS One. 2015;10: e0120504 10.1371/journal.pone.0120504 25781328 PMC4362943

[17] Yano T, Moe KK, Yamazaki K, Ooka T, Hayashi T, Misawa N. Identification of candidate pathogens of papillomatous digital dermatitis in dairy cattle from quantitative 16S rRNA clonal analysis. Vet Microbiol. 2010;143: 352–362. 10.1016/j.vetmic.2009.12.009 20036086

[18] Santos TM, Pereira RV, Caixeta LS, Guard CL, Bicalho RC. Microbial diversity in bovine papillomatous digital dermatitis in Holstein dairy cows from upstate New York. FEMS Microbiol Ecol. 2012;79: 518–529. 10.1111/j.1574-6941.2011.01234.x 22093037

[19] Klitgaard K, Nielsen MW, Ingerslev HC, Boye M, Jensen TK. Discovery of bovine digital dermatitis-associated Treponema spp. in the dairy herd environment by a targeted deep-sequencing approach. Appl Environ Microbiol. 2014;80: 4427–4432. 10.1128/AEM.00873-14 24814794 PMC4068665

[20] Shah N, Tang H, Doak TG, Ye Y. Comparing bacterial communities inferred from 16S rRNA gene sequencing and shotgun metagenomics. Pac Symp Biocomput. 2011: 165–176. 21121044 10.1142/9789814335058_0018

[21] Meyer F, Paarmann D, D'Souza M, Olson R, Glass EM, Kubal M, et al The metagenomics RAST server—a public resource for the automatic phylogenetic and functional analysis of metagenomes. BMC Bioinformatics. 2008;9: 386-2105-9-386. 10.1186/1471-2105-9-386 PMC2563014 18803844

[22] Storey JD. A direct approach to false discovery rates. Journal of the Royal Statistical Society: Series B (Statistical Methodology, 64: 479–498. 10.1111/1467-9868.00346 2002.

[23] Kalyuzhnaya MG, Lapidus A, Ivanova N, Copeland AC, McHardy AC, Szeto E, et al High-resolution metagenomics targets specific functional types in complex microbial communities. Nat Biotechnol. 2008;26: 1029–1034. 10.1038/nbt.1488 18711340

[24] Kalyuzhnaya MG, Lidstrom ME, Chistoserdova L. Real-time detection of actively metabolizing microbes by redox sensing as applied to methylotroph populations in Lake Washington. ISME J. 2008;2: 696–706. 10.1038/ismej.2008.32 18607374

[25] Evans NJ, Brown JM, Demirkan I, Murray RD, Vink WD, Blowey RW, et al Three unique groups of spirochetes isolated from digital dermatitis lesions in UK cattle. Vet Microbiol. 2008;130: 141–150. 10.1016/j.vetmic.2007.12.019 18243592

[26] Lux R, Moter A, Shi W. Chemotaxis in pathogenic spirochetes: directed movement toward targeting tissues? J Mol Microbiol Biotechnol. 2000;2: 355–364. 11075906

[27] Li C, Motaleb A, Sal M, Goldstein SF, Charon NW. Spirochete periplasmic flagella and motility. J Mol Microbiol Biotechnol. 2000;2: 345–354. 11075905

[28] Thomas DD, Navab M, Haake DA, Fogelman AM, Miller JN, Lovett MA. Treponema pallidum invades intercellular junctions of endothelial cell monolayers. Proc Natl Acad Sci U S A. 1988;85: 3608–3612. 3285346 10.1073/pnas.85.10.3608 PMC280263

[29] Comstock LE, Thomas DD. Penetration of endothelial cell monolayers by Borrelia burgdorferi. Infect Immun. 1989;57: 1626–1628. 2707862 10.1128/iai.57.5.1626-1628.1989 PMC313325

[30] Peters SR, Valdez M, Riviere G, Thomas DD. Adherence to and penetration through endothelial cells by oral treponemes. Oral Microbiol Immunol. 1999;14: 379–383. 10895695 10.1034/j.1399-302x.1999.140609.x

[31] Lux R, Miller JN, Park NH, Shi W. Motility and chemotaxis in tissue penetration of oral epithelial cell layers by Treponema denticola. Infect Immun. 2001;69: 6276–6283. 11553571 10.1128/IAI.69.10.6276-6283.2001 PMC98762

[32] Maruyama M, Lodderstaedt G, Schmitt R. Purification and biochemical properties of complex flagella isolated from Rhizobium lupini H13-3. Biochim Biophys Acta. 1978;535: 110–124. 667114 10.1016/0005-2795(78)90038-7

[33] Trachtenberg S, DeRosier DJ, Macnab RM. Three-dimensional structure of the complex flagellar filament of Rhizobium lupini and its relation to the structure of the plain filament. J Mol Biol. 1987;195: 603–620. 3656426 10.1016/0022-2836(87)90185-9

[34] Li C, Wolgemuth CW, Marko M, Morgan DG, Charon NW. Genetic analysis of spirochete flagellin proteins and their involvement in motility, filament assembly, and flagellar morphology. J Bacteriol. 2008;190: 5607–5615. 10.1128/JB.00319-08 18556797 PMC2519375

[35] Sim JH, Shi W, Lux R. Protein-protein interactions in the chemotaxis signalling pathway of Treponema denticola. Microbiology. 2005;151: 1801–1807. 15941989 10.1099/mic.0.27622-0

[36] Weinberg ED. Iron and infection. Microbiol Rev. 1978;42: 45–66. 379572 10.1128/mr.42.1.45-66.1978 PMC281418

[37] DiGuiseppi J, Fridovich I. Oxygen toxicity in Streptococcus sanguis. The relative importance of superoxide and hydroxyl radicals. J Biol Chem. 1982;257: 4046–4051. 6279624

[38] Neilands JB. Microbial iron compounds. Annu Rev Biochem. 1981;50: 715–731. 6455965 10.1146/annurev.bi.50.070181.003435

[39] Neilands JB, Erickson TJ, Rastetter WH. Stereospecificity of the ferric enterobactin receptor of Escherichia coli K-12. J Biol Chem. 1981;256: 3831–3832. 6452456

[40] Brooks BE, Buchanan SK. Signaling mechanisms for activation of extracytoplasmic function (ECF) sigma factors. Biochim Biophys Acta. 2008;1778: 1930–1945. 17673165 10.1016/j.bbamem.2007.06.005 PMC2562455

[41] Speijers MH, Baird LG, Finney GA, McBride J, Kilpatrick DJ, Logue DN, et al Effectiveness of different footbath solutions in the treatment of digital dermatitis in dairy cows. J Dairy Sci. 2010;93: 5782–5791. 10.3168/jds.2010-3468 21094750

[42] Thomsen PT. Short communication: Efficacy of copper sulfate hoof baths against digital dermatitis-Where is the evidence? J Dairy Sci. 2015. 10.3168/jds.2014-9135 25622864

[43] Zuerner RL, Heidari M, Elliott MK, Alt DP, Neill JD. Papillomatous digital dermatitis spirochetes suppress the bovine macrophage innate immune response. Vet Microbiol. 2007;125: 256–264. 17628359 10.1016/j.vetmic.2007.06.001

[44] Dopfer D, Anklam K, Mikheil D, Ladell P. Growth curves and morphology of three Treponema subtypes isolated from digital dermatitis in cattle. Vet J. 2012;193: 685–693. 10.1016/j.tvjl.2012.06.054 22901455

[45] Gomez A, Anklam KS, Cook NB, Rieman J, Dunbar KA, Cooley KE, et al Immune response against Treponema spp. and ELISA detection of digital dermatitis. J Dairy Sci. 2014;97: 4864–4875. 10.3168/jds.2013-7616 24931522

[46] Trott DJ, Moeller MR, Zuerner RL, Goff JP, Waters WR, Alt DP, et al Characterization of Treponema phagedenis-like spirochetes isolated from papillomatous digital dermatitis lesions in dairy cattle. J Clin Microbiol. 2003;41: 2522–2529. 12791876 10.1128/JCM.41.6.2522-2529.2003 PMC156514

[47] Berry SL, Read DH, Famula TR, Mongini A, Dopfer D. Long-term observations on the dynamics of bovine digital dermatitis lesions on a California dairy after topical treatment with lincomycin HCl. Vet J. 2012;193: 654–658. 10.1016/j.tvjl.2012.06.048 22892182

[48] Schmitz-Esser S, Toenshoff ER, Haider S, Heinz E, Hoenninger VM, Wagner M, et al Diversity of bacterial endosymbionts of environmental acanthamoeba isolates. Appl Environ Microbiol. 2008;74: 5822–5831. 10.1128/AEM.01093-08 18641160 PMC2547052

[49] Schmitz-Esser S, Tischler P, Arnold R, Montanaro J, Wagner M, Rattei T, et al The genome of the amoeba symbiont "Candidatus Amoebophilus asiaticus" reveals common mechanisms for host cell interaction among amoeba-associated bacteria. J Bacteriol. 2010;192: 1045–1057. 10.1128/JB.01379-09 20023027 PMC2812958

