# Supplementary figures and images for: Shotgun Metagenomic Sequencing Reveals Functional Genes and Microbiome Associated with Bovine Digital Dermatitis
Source: PLoS One. 2015 Jul 20;10(7):e0133674. doi: 10.1371/journal.pone.0133674 (PMC4508036; doi:10.1371/journal.pone.0133674)

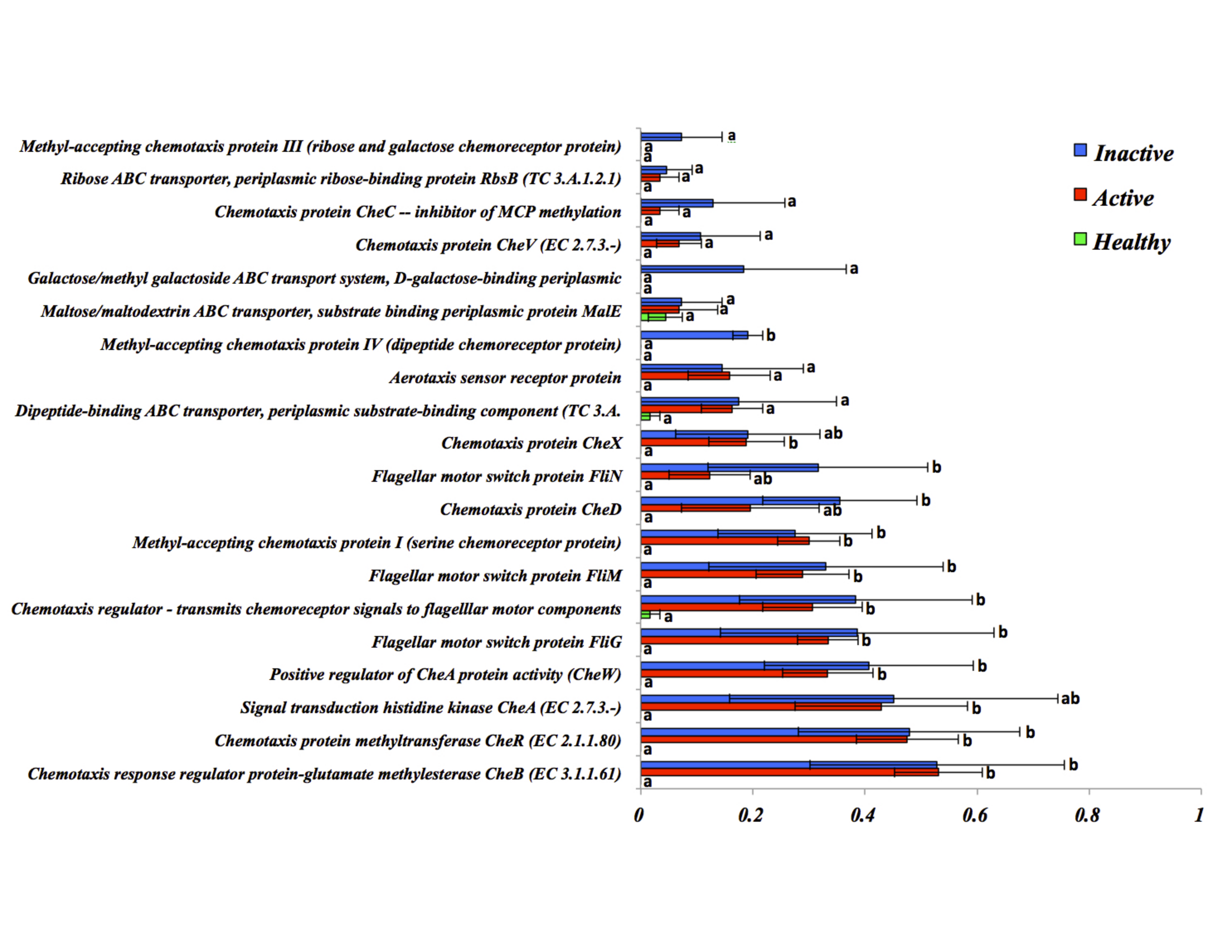

Supplement: S1 Fig — Error bars represented the standard error of the mean. Different letters means P < 0.05. (TIF) [file pone.0133674.s001.tif]

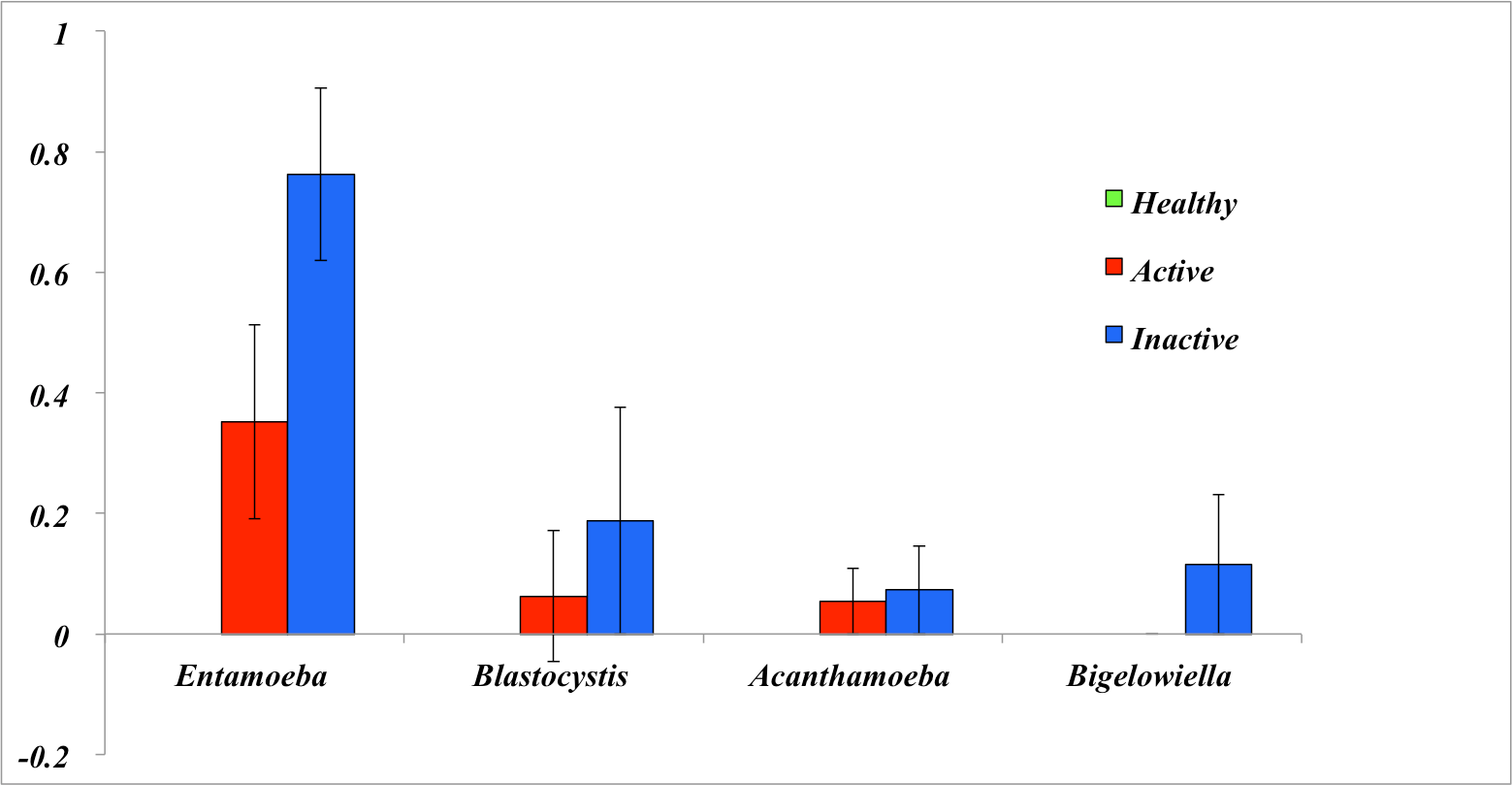

Supplement: S2 Fig — Error bars represent standard error of the mean. (TIF) [file pone.0133674.s002.tif]

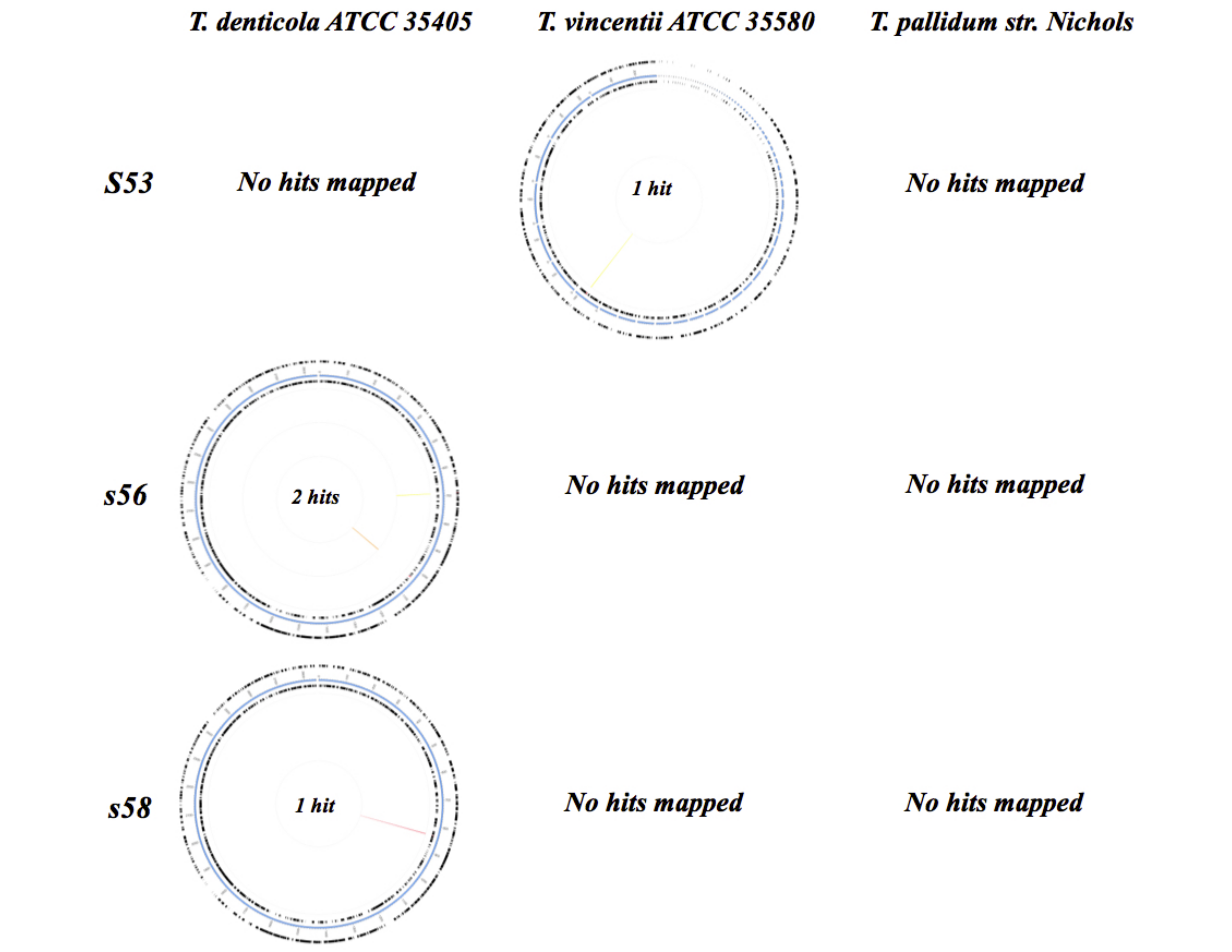

Supplement: S3 Fig — The blue circle represents the bacterial contigs for the genome of interest, while the two black rings map genes on the forward and reverse strands. Bars represent hits distributions and the colors are coded according to the e-value of the matches with red (-30 and less), orange (-20 to -30), yellow (-10 to -20), green (-5 to -10) and blue (-3 to -5). (TIF) [file pone.0133674.s003.tif]
