# Supplementary material for: Shotgun Metagenomic Sequencing Reveals Functional Genes and Microbiome Associated with Bovine Digital Dermatitis
Source: PLoS One. 2015 Jul 20;10(7):e0133674. doi: 10.1371/journal.pone.0133674 (PMC4508036; doi:10.1371/journal.pone.0133674)
Supplement: S1 Table — (PDF) [file pone.0133674.s005.pdf]

	Mean (95% Confidence Interval)			
Flagellum Proteins	HBS	ADD	IDD	*p*-value
Fla protein FlaA	0	0.508(0.67,0.35)	0.500(1.07,-0.07)	0.0029
RNA polymerase sigma factor RpoD	0.083(0.15,0.02)	0.401(0.59,0.22)	0.507(0.91,0.10)	0.0085
Fla hook-associated protein FlgK	0	0.411(0.57,0.25)	0.466(0.83,0.10)	0.0006
Fla biosynthesis protein FlhA	0	0.393(0.55,0.24)	0.462(0.88,0.04)	0.0015
Fla motor switch protein FliG	0	0.351(0.46,0.24)	0.405(0.91,-0.10)	0.0084
RNA polymerase sigma-54 factor RpoN	0	0.427(0.56,0.30)	0.312(0.64,-0.02)	0.0005
Fla hook protein FlgE	0	0.288(0.37,0.21)	0.445(0.81,0.08)	0.0008
Fla-specific ATP synthase FliI	0.018(0.05,-0.02)	0.301(0.42,0.18)	0.406(0.91,-0.10)	0.0202
Fla M-ring protein FliF	0	0.309(0.45,0.17)	0.398(0.93,-0.03)	0.0067
Fla motor rotation protein MotA	0.018(0.05,-0.02)	0.303(0.48,0.13)	0.378(0.83,-0.08)	0.0218
Fla motor switch protein FliM	0	0.303(0.48,0.13)	0.348(0.78,-0.08)	0.0144
Fla biosynthesis protein FlhB	0	0.305(0.53,0.08)	0.336(0.82,-0.04)	0.0157
Fla motor rotation protein MotB	0	0.279(0.55,0.01)	0.359(0.78,-0.06)	0.0346
Fla basal-body rod protein FlgG	0	0.307(0.43,0.18)	0.260(0.77,-0.25)	0.0441
Fla hook-associated protein FliD	0	0.181(0.25,0.11)	0.381(0.62,0.14)	0.0002
Fla hook-associated protein FlgL	0	0.217(0.40,0.03)	0.330(0.70,-0.04)	0.0216
Fla biosynthesis protein FliP	0	0.274(0.39,0.16)	0.269(0.60,-0.06)	0.0069
RNA polymerase sigma factor for fla operon	0	0.213(0.36,0.07)	0.301(065,-0.05)	0.0169
Fla basal-body rod modification protein FlgD	0.018(0.05,-0.02)	0.223(0.40,0.05)	0.265(0.59,-0.06)	0.0436
Fla basal-body rod protein FlgF	0	0.228(0.40,0.03)	0.266(0.66,-0.12)	0.0499
Fla motor switch protein FliN	0	0.129(0.28,-0.02)	0.333(0.74,-0.07)	0.0334
Fla assembly protein FliH	0	0.187(0.34,0.03)	0.273(0.61,-0.06)	0.0298

Fla basal-body rod protein FlgC	0	0.187(0.41,-0.04)	0.241(0.58,-0.10)	0.0854
Fla synthesis regulator FleN	0	0.181(0.36,0.00)	0.245(0.59,-0.10)	0.0628
Fla biosynthesis protein FliR	0	0.129(0.30,-0.04)	0.253(0.62,-0.11)	0.0870
Fla biosynthesis protein FliS	0	0.129(0.22,0.04)	0.208(0.49,-0.07)	0.0357
Fla hook-basal body complex protein FliE	0	0.129(0.30,-0.04)	0.160(0.35,-0.03)	0.0819
Fla protein FlbB	0	0.108(0.24,-0.03)	0.173(0.51-0.17)	0.1874
Fla basal-body rod protein FlgB	0	0.166(0.21,0.12)	0.112(0.33,-0.11)	0.0153
Fla biosynthesis protein FliQ	0	0.108(0.24,-0.03)	0.153(0.45,-0.15)	0.1856
Fla protein FlbD	0	0.072(0.15,-0.01)	0.160(0.35,-0.03)	0.0346
Fla hook-length control protein FliK	0.036(0.08,-0.01)	0.057(0.17,-0.05)	0.135(0.40,-0.13)	0.5091
Fla biosynthesis protein FlhF	0	0.000	0.208(0.49,-0.07)	0.0243
Fla protein FlgJ [peptidoglycan hydrolase]	0	0.093(0.20,-0.02)	0.096(0.19,0.00)	0.0514
Fla protein FlaB	0	0.036(0.11,-0.03)	0.153(0.45,-0.15)	0.1827
Fla P-ring protein FlgI	0	0.036(0.11,-0.03)	0.124(0.37,-0.12)	0.1968
Fla protein FlaG	0	0.072(0.15,-0.01)	0.048(0.14,-0.05)	0.1122
Flag regulatory protein FleQ	0	0.036(0.11,-0.03)	0.048(0.14,-0.05)	0.2981
Fla biosynthesis protein FliL	0	0.072(0.15,-0.01)	0	0.0369
Fla biosynthesis protein FliZ	0	0.036(0.11,-0.03)	0	0.2692
Fla protein FliJ	0	0.036(0.11,-0.03)	0	0.2692
